# Conference Summary

**DOI:** 10.3201/eid0801.01-0411

**Published:** 2002-01

**Authors:** Charles Calisher, Colorado State University, Fort Collins, Colorado

**Affiliations:** Antti Vaheri, Haartman Institute, Helsinki, Finland

The Fifth International Conference on Hemorrhagic Fever with Renal Syndrome, Hantavirus Pulmonary Syndrome, and Hantaviruses was held June 13-16, 2001, in Annecy, a scenic resort in the French Alps. The conference, with 160 participants, was co-organized and generously hosted by the Mérieux Foundation.

Apart from Eurasia and the Americas, evidence for infections with hantaviruses now has been reported from many new areas, including the Southeast Asian countries of Cambodia, Indonesia, Taiwan, and Thailand. However, definitive and consistent evidence for the presence of these viruses still has not been reported from Africa or Australia. The occurrence of some newly recognized pathogenic Old World hantaviruses was reported (Amur virus from *Apodemus peninsulae* in the Far East and Saaremaa virus from *A. agrarius* in Europe). For the Old World, the proper taxonomy of these viruses seems to be reasonably well established. This is not the case for South American hantaviruses, presumably because rodents and their hantaviruses only recently entered the area, perhaps after the opening of the "Panama Bridge," as summarized by S. Morzunov (Reno, NV). The clinical pictures of South American hantavirus infections (D. Enria, Pergamino, Argentina) seem to be variable, intermediate between severe North American hantavirus pulmonary syndrome (HPS) and Eurasian hemorrhagic fever with renal syndrome (HFRS), and include mild cases, hemorrhagic cases, and those characterized by renal and neurologic signs**.**Additional evidence was presented for the occurrence of clustered cases, further suggesting the possibility of person-to-person transmission, in southern Argentina, Chile, and Brazil. Much additional work is required to prove such a hypothesis.

Transmission of hantaviruses in carrier rodents is complex and may be attributable to decreased biodiversity of rodent reservoir communities (J. Mills, Atlanta, GA). Although it is well known that wounds and infections are correlated, deer mice in laboratory colonies shed very little *Sin Nombre virus_ *(SNV). According to S. Klein (Baltimore, MD), intact male rats shed more *Seoul virus* than do females or castrated males. As reported by B. Hjelle (Albuquerque, NM), heat shock in cell cultures and cold shock in vivocan reactivate SNV, which persists in heart, lung, and, interestingly, brown fat.

The pathogenesis of hantavirus infections is not well understood. Some progress was reported towards establishing reverse genetics for hantaviruses, as the catalytic core domain of *Hantaan virus* (HV) RNA polymerase has been isolated (C. Jonsson, Las Cruces NM). Also of special interest was the report by E. Mackow (Stony Brook, NY), on the selective inhibition of beta3-integrin directed endothelial cell migration by pathogenic hantaviruses. Cytotoxic T cells may also play an important role in affecting vascular permeability in HFRS and HPS (H. Van Epps and F. Ennis, Worcester, MA). Interestingly, while HFRS caused by *Puumala virus* may lead to increased blood pressure (J. Mustonen, Tampere, Finland) and HPS by SNV to pulmonary sequelae (D. Goade, Albuquerque, NM) as long-term effects, both HFRS and HPS can be characterized by increased proteinuria as a late consequence. Several reports on multiple cytokine mRNA and protein responses in cultured cells and in patients were, to say the least, somewhat contradictory; more work is needed to clarify these phenomena.

Classical Salk-type hantavirus vaccines have been widely and successfully used for a number of years in Korea and China. Meanwhile, more and more sophisticated recombinant and DNA vaccines are being developed in Europe and North America, but none has entered the market. This work is, however, producing highly interesting results, such as the *Andes virus* lethal model in adult hamsters (J. Hooper, Frederick, MD) and the recombinant human antibodies with good therapeutic potential (J. Koch, Heidelberg, Germany).

In summary, the 3 days in Annecy provided a comprehensive progress report of the field, one that may be summarized by Mark Twain's words, "Interesting if true, interesting anyway." The participants of the conference were confident enough to found "The International Society for Hantaviruses and Hantaviral Diseases" and elected Ho Wang Lee (Seoul, Korea), the discoverer of HV, the prototype hantavirus, as its first president.

_

